# Efficient, Rapid, and Sensitive Detection of Plant RNA Viruses With One-Pot RT-RPA–CRISPR/Cas12a Assay

**DOI:** 10.3389/fmicb.2020.610872

**Published:** 2020-12-17

**Authors:** Rashid Aman, Ahmed Mahas, Tin Marsic, Norhan Hassan, Magdy M. Mahfouz

**Affiliations:** Laboratory for Genome Engineering and Synthetic Biology, Division of Biological Sciences, King Abdullah University of Science and Technology, Thuwal, Saudi Arabia

**Keywords:** RT-RPA, CRISPR-Cas12a, plant virus RNA, biosensors, diagnostics

## Abstract

Most viruses that infect plants use RNA to carry their genomic information; timely and robust detection methods are crucial for efficient control of these diverse pathogens. The RNA viruses, potexvirus (*Potexvirus*, family *Alphaflexiviridae*), potyvirus (*Potyvirus*, family *Potyviridae*), and tobamovirus (*Tobamovirus*, family *Virgaviridae*) are among the most economically damaging pathogenic plant viruses, as they are highly infectious and distributed worldwide. Their infection of crop plants, alone or together with other viruses, causes severe yield losses. Isothermal nucleic acid amplification methods, such as loop-mediated isothermal amplification (LAMP), recombinase polymerase amplification (RPA), and others have been harnessed for the detection of DNA- and RNA-based viruses. However, they have a high rate of non-specific amplification and other drawbacks. The collateral activities of clustered regularly interspaced short palindromic repeats (CRISPR) and CRISPR-associated nuclease Cas systems such as Cas12 and Cas14 (which act on ssDNA) and Cas13 (which acts on ssRNA) have recently been exploited to develop highly sensitive, specific, and rapid detection platforms. Here, we report the development of a simple, rapid, and efficient RT- RPA method, coupled with a CRISPR/Cas12a-based one-step detection assay, to detect plant RNA viruses. This diagnostic method can be performed at a single temperature in less than 30 min and integrated with an inexpensive commercially available fluorescence visualizer to facilitate rapid, in-field diagnosis of plant RNA viruses. Our developed assay provides an efficient and robust detection platform to accelerate plant pathogen detection and fast-track containment strategies.

## Introduction

Despite the significant increase of ssDNA viruses in recent years, RNA viruses are still the most prevalent disease-causing agents in plants and pose a major threat to agriculture and food security. Most plant RNA viruses have a plus single-stranded RNA (+ ssRNA) genome that encodes the proteins required for replication, translation, encapsidation, cell to cell movement, and others (Joshi and Haenni, 1984). Their genomes take on the role of messenger RNA (mRNA) in host cells and are translated into functional proteins that are involved, directly or indirectly, in virus replication. Plant RNA viruses with a negative double-stranded RNA (-dsRNA) or negative single-stranded RNA (-ssRNA) genome are first transcribed and the resulting RNAs are then translated into functional viral proteins (Joshi and Haenni, 1984). The + ssRNA-based viruses potato virus X (PVX), potato virus Y (PVY), and tobacco mosaic virus (TMV) are among the most economically important viruses based on their worldwide distribution and effect on crop yield and quality (Scholthof et al., 2011; Rybicki, 2015). PVX predominantly spreads via mechanical transmission and causes 10–15% yield loss in potato (*Solanum tuberosum*) (Kreuze et al., 2020). Its symptoms vary from weak to intense, resulting in smaller leaves and stunted plants. PVY is transmitted by over 25 different aphid species and can also be mechanically transmitted, and poses a significant threat to tuber yield and quality as well as seed production of potato worldwide (Lawson et al., 1990; Kreuze et al., 2020). TMV, the iconic first recognized virus, also spreads via mechanical transmission and is of major economic importance, as TMV infections cause mottled browning of tobacco (*Nicotiana tabacum*) leaves (Hadidi et al., 2020). TMV also infects other Solanaceous crops plants, particularly tomato (*Solanum lycopersicum*) (Rifkind and Freeman, 2005; Kumar et al., 2011).

The development of a robust and sensitive diagnostic method is of utmost importance to control plant viruses. Several methods have been developed, including, but not limited to, enzyme-linked immunosorbent assay (ELISA), molecular hybridization, polymerase chain reaction (PCR), reverse transcription followed by PCR (RT-PCR), loop (or RT-loop) mediated isothermal amplifications (LAMP and RT-LAMP), and microarrays (Baranwal et al., 2020; Hadidi et al., 2020; Rubio et al., 2020). ELISA depends on binding viral-specific antibodies to the virus coat protein (Clark and Adams, 1977). Molecular hybridization techniques like Southern and northern blotting use sequence-specific probes to identify DNA or RNA molecules, respectively (Hadidi et al., 2020), that can be later visualized by different methods, including simple chemiluminescent detection. PCR, RT-PCR, RT-LAMP, and LAMP, which do not require cycling of temperature, in contrast to PCR, have further streamlined plant pathogens detection (Faggioli et al., 2017). Real-time quantitative PCR (qPCR) and (RT-qPCR) rely on the binding of a virus sequence-specific fluorescent probe to the PCR-amplified region and are conventional techniques used for the detection of DNA and RNA viruses (Lin et al., 2013; Faggioli et al., 2017; Jarošová et al., 2018). Although widely used, qPCR and RT-qPCR detection methods require well-equipped laboratories and trained personnel, impeding the use of these technologies at point-of-care or resource-limited areas.

By contrast, isothermal amplification techniques such as helicase-dependent amplification (HDA), nucleic acid sequence-based amplification (NASBA), LAMP, and recombinase polymerase amplification (RPA) allow nucleic acid amplification at a single temperature, thus supporting their use in the field for on-site diagnosis in a low-resource environment (Compton, 1991; Notomi et al., 2000; Vincent et al., 2004; Piepenburg et al., 2006; Rubio et al., 2020). LAMP and RPA DNA amplification approaches have been further customized as RT-LAMP and RT-RPA when combined with a suitable reverse transcriptase for pathogens harboring an RNA genome. RT-LAMP and RT-RPA have been utilized for the detection of many plant viruses, including PVX and PVY (Fukuta et al., 2003; Nie, 2005; Euler et al., 2013; Uehara-Ichiki et al., 2013; Budziszewska et al., 2016; Faggioli et al., 2017; Silva et al., 2018). Although these isothermal amplification methods are highly sensitive, they also demand additional care to minimize non-specific amplification that often results in false-positive results.

Clustered regularly short palindromic repeat-associated systems (CRISPR-Cas) are derived from a natural immune-like system that provides molecular immunity to microorganisms (bacterial and archaeal species) against foreign invading species. CRISPR-Cas has been extensively exploited in eukaryotic species for genome engineering, molecular immunity, and transcriptome regulation (Ran et al., 2013; Hsu et al., 2014; Ali et al., 2015; Aman et al., 2018; Mahas et al., 2019). In several CRISPR systems target identification and cleavage results in the activation of their non-specific endonuclease activity (Aman et al., 2020), which has been harnessed for nucleic acid detection. For instance, the well-known nucleic acid detection platforms SHERLOCK (Specific High-sensitivity Enzymatic Reporter UnLOCKing) and DETECTR (DNA Endonuclease-Targeted CRISPR Trans Reporter) employ the collateral activities of Cas13 and Cas12, respectively, when using ssRNA or ssDNA reporters (Chen et al., 2018; Gootenberg et al., 2018; Li et al., 2018a,b; Kellner et al., 2019). End-point or quantitative measurements can be obtained by using a variety of fluorophore- or biotin-labeled reporters.

LAMP and RPA methods have been successfully integrated with CRISPR/Cas technology for rapid and portable detection of viral nucleic acids (Kellner et al., 2019; Broughton et al., 2020). We recently developed the ***i****n vitro* **S**pecific **C**RISPR-based **A**ssay for **N**ucleic acids detection (iSCAN) for sensitive and rapid detection of SARS-CoV-2, the causal agent of COVID-19, which combines the target sequence amplification via RT-LAMP with the specific detection and subsequent collateral activity of CRISPR/Cas12 (Ali et al., 2020). To mitigate the non-specific amplification and cross-contamination issues inherent in these amplification methods, one-pot assays have been recently developed for virus detection (Joung et al., 2020). In this study, we have applied iSCAN, after replacing RT-LAMP with RT-RPA, to develop a one-pot detection assay named as iSCAN-one-pot (iSCAN-OP), for the detection of plant RNA viruses. We report that our developed iSCAN-OP is simple, specific, rapid, and sensitive for the detection of plant RNA viruses. The RT-RPA pre-amplification step converts the RNA genome of the virus into dsDNA that serves as a substrate for the Cas12a cis activity. Cas12a targeting of the dsDNA triggers its collateral activity, which in turn cleaves the ssDNA reporter molecules and releasing the signal (Chen et al., 2018; Ding et al., 2020). We further combined the iSCAN-OP detection assay with a commercially available P51 fluorescence viewer device to facilitate quick, affordable, in-field diagnosis of plant RNA viruses. Our iSCAN-OP detection assay can be easily adapted for large-scale virus screenings in the field and will constitute a critical tool in agriculture biotechnology for rapid diagnostics of plant diseases.

## Materials and Methods

### Nucleic Acid Preparation

For *in vitro*-transcribed viral RNA, the coding sequence for the coat protein (CP) of each virus was amplified via PCR with the appropriate virus-specific oligonucleotides (Supplementary Table S1). PCR products were then purified using a QIAquick PCR purification kit as per the manufacturer’s protocol. A total of 1–1.5 μg of each PCR product was *in vitro*-transcribed at 37°C overnight (8 h) with the TranscriptAid T7 High Yield Transcription Kit (Thermo Fisher Scientific K0441). *In vitro*-transcribed viral RNA was then purified using the DIRECTZOL KIT from ZymoBiomics (Direct-Zol RNA Miniprep kit; catalog #R2070) following the manufacturer’s protocol. For real viral samples, 3-week-old *Nicotiana benthamiana* plants were infected with the virus, as previously described (Aman et al., 2018). Total plant RNA was extracted from systemic leaves 7–10 days after infiltration (dai) using the DIRECTZOL KIT from ZymoBiomics (Direct-Zol RNA Miniprep kit; catalog #R2070). The concentrations of *in vitro* transcribed RNA and total plant RNA templates used in the RT-RPA reactions were adjusted to 100 ng/μL with sterile water.

All CRISPR RNAs (crRNAs) were produced by *in vitro* transcription of commercially synthesized ssDNA oligos with an appended T7 promoter sequence (Sigma), as previously reported (Abudayyeh et al., 2019; Supplementary Table S2). The synthetized ssDNAs were annealed to a forward-directed T7 promoter primer (Sigma) to initiate *in vitro* transcription of crRNAs at 37°C overnight (8 h). All crRNAs were purified using the DIRECTZOL KIT from ZymoBiomics.

### Cas12 Protein Purification and Assessment of Activity

LbCas12a protein from *Lachnospiraceae bacterium* (LbCas12a) was purified as previously described by Chen et al. (2018) and Ali et al. (2020) protocol. The target-specific endonuclease activity of Cas12a was tested and validated against a PCR product amplified from the PVX, PVY, and TMV CP coding sequences, as previously described (Ali et al., 2020). The collateral *trans* cleavage activity of LbCas12a was measured by providing the Hex-labeled ssDNA reporter (/5HEX/TTTTTTT/3IABkFQ/) in the restriction reaction. The fluorescence signal as a result of trans-cleavage activity of Cas12a was measured with the P51 Molecular Fluorescence Viewe^1^.

### Design and Screening of RPA Primers

For each virus template (TMV NCBI ID: MK087763.1, PVX NCBI ID: M95516.1 and PVY NCBI ID: HM367076.1), 3–4 sets of RPA primers (Supplementary Table S3) were designed as per the manufacturer’s instructions to amplify a region of 100–200 bp. The length and melting temperatures of the primers varied from 30 to 35 bases and 54–67°C, respectively. All primers used in RPA assays were from Sigma. The efficiency of different sets of RPA primers was tested with *in vitro*-transcribed viral RNA, and the signal was examined using a Tecan plate reader (M200) or P51 Molecular Fluorescence Viewer.

### iSCAN-Two-Pot (iSCAN-TP) Detection Assay

**FIGURE 1:**
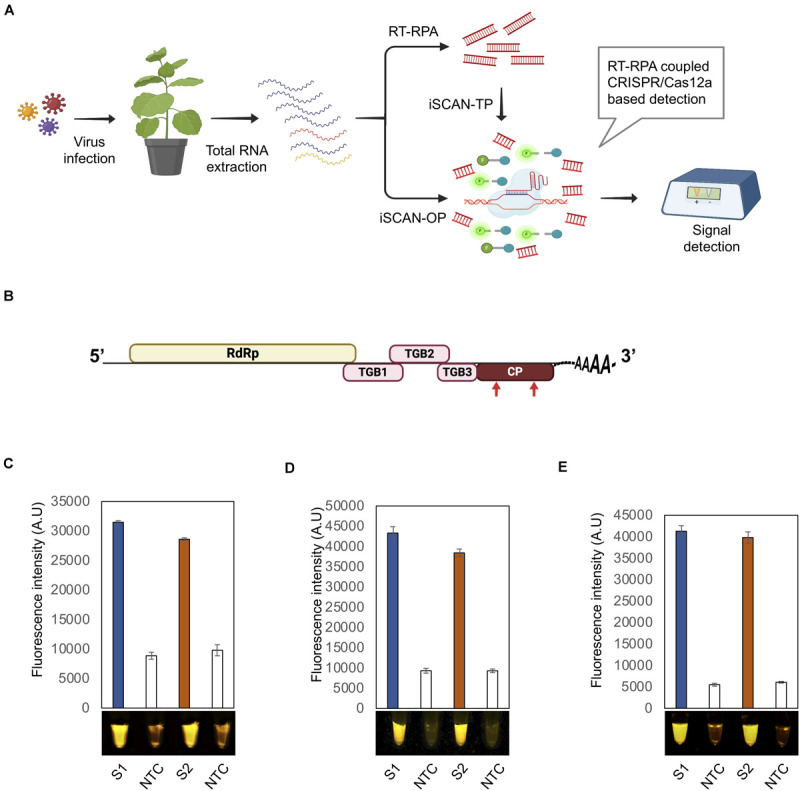
Optimization of the iSCAN-OP detection assay for PVX and PVY detection**. (A)** Workflow of RT-RPA coupled CRISPR/Cas12a based detection system. **(B)** A schematic view of the potato virus X (PVX) genome. Red arrows indicate the regions targeted by the RT-RPA coupled CRISPR/Cas12a based detection system. **(C)** End-point fluorescence visualization of the iSCAN-TP detection assay (*in vitro* transcribed RNA) with P51 Molecular Fluorescence Viewer. S1: Primer set 1, S2: Primer set 2, NTC: no template control. Values are shown in the graph as means ± SD (*n* = 3). **(D)** End-point fluorescence visualization of the iSCAN-OP detection assay (*in vitro* transcribed RNA) with P51 Molecular Fluorescence Viewer. S1: Primer set 1, S2: Primer set 2, NTC: no template control. Values are shown in the graph as means ± SD (*n* = 3). **(E)** End-point fluorescence visualization of the iSCAN-OP detection assay (*in vitro* transcribed RNA) with P51 Molecular Fluorescence Viewer. S1: Primer set 1, S2: Primer set 2, NTC: no template control. Values are shown in the graph as means ± SD (*n* = 3).

Total RNA extracted from infected plants was subjected to iSCAN-TP or iSCAN one-pot (iSCAN-OP) before being handed over to CRISPR/Cas-based detection in a two- or one-pot reaction, respectively. The fluorescent signal resulting from Cas12a-based collateral cleavage of the single-stranded DNA-fluorescent quencher (ssDNA-FQ) reporter was visualized via a fluorescence viewer (Figure 1A).

The iSCAN-TP detection assays were performed in two steps. The RT-RPA assay was carried out according to the manufacturer’s protocol (TwistAmp^TM^ Basic Kit “Improved Formulation,” cat. no. TABAS03KIT) with slight modifications. Briefly, first resuspending the RPA pellet with 29.5 μL resuspension buffer, then 11.8 μL of the resuspended RPA mixture was mixed with 0.5 μL Superscript III Reverse Transcriptase, 0.5 μL forward primer (10 μM), 0.5 μL reverse primer (10 μM), 1 μL *in vitro* transcribed or total plant RNA (100 ng) and 0.2 μL nuclease-free water. Reactions tubes were mixed thoroughly by pipetting, before adding 2 μL of 280 nM magnesium acetate. The tubes were incubated at 42°C to start the RT-RPA reaction. Next, for Cas12 based detection assays, 250 nM of LbCas12a protein was first pre-incubated with 250 nM of LbCas12a crRNAs in 1x Cas12 reaction Buffer (20 mM HEPES (pH 7.5), 150 mM KCl, 10 mM MgCl2, 1% glycerol and 0.5 mM DTT) for 30 min at 37°C to assemble Cas12-crRNA ribonucleoprotein (RNP) complexes as previously described (Chen et al., 2018). The reaction was diluted 4 times with 1x binding buffer (20 mM Tris-HCl, pH 7.5, 100 mM KCl, 5 mM MgCl2, 1 mM DTT, 5% glycerol, 50 μg mL-1 heparin) (Chen et al., 2018). Two microliter aliquot of the RT-RPA products was added to a pre-assembled and diluted Cas12a/crRNA ribonucleoprotein (RNP) complex with the addition of the Hex-labeled ssDNA reporter for fluorescence-based detection. The reaction was then incubated for another 15–30 min at 37°C and the fluorescent signal was monitored using either a Tecan plate reader (M200) or a P51 Molecular Fluorescence Viewer.

### iSCAN-OP Detection Assay

The difference between iSCAN one-pot and two-pot assays is that, in two-pot, the target sequence is amplified first by isothermal amplification methods such as RPA in a separate tube. The amplified product is then added into another separate tube that has the detection reagents such as Cas12a, crRNA and the reporter. In case of one-pot assays, both steps (target sequence amplification and Cas12a-based detection) are performed in the same tube at the same temperature (Kellner et al., 2019).

The iSCAN-OP detection assays were performed by first resuspending the RPA pellet with 29.5 μL resuspension buffer, followed by the addition of 1 μL Superscript III Reverse Transcriptase (Invitrogen), 1.5 μL forward primer (10 μM), 1.5 μL reverse primer (10 μM), 2 μL Cas12a (5 μM), 2 μL crRNA (5 μM), 2.5 μL nuclease-free water, and 4 μL Hex reporter (10 μM). Reaction tubes were mixed by pipetting, and the mixture was divided equally 22 μL into two tubes. 1 μL of synthetic or total plant RNA (100 ng) and 2 μL of 280 mM magnesium acetate was then added to the reaction tube and incubated at 42°C for 20 min. The end-point fluorescence signal was monitored using either a Tecan plate reader (M200) or a P51 Molecular Fluorescence Viewer.

### Real-Time Detection of *in vitro* Transcribed TMV RNA

To determine the sensitivity of RT-RPA-CRISPR/Cas12a detection method, we diluted *in vitro* transcribed TMV RNA down to femtomolar concentrations and set up one-pot RT-RPA as described above, with the addition of 250 nM FAM reporter (5′-/56-FAM/TTATT/3IABKFQ/-3′, IDT) instead of HEX reporter. To detect the FAM signal in real-time, the reaction samples were run in an Applied Biosystems StepOne Real-time PCR system for 1 h at 42°C with fluorescent measurements every 2 min. As a no-template control, we added water instead of TMV synthetic RNA.

## Results

### Optimization of the iSCAN-OP Detection Assay for PVX and PVY Detection

To test the activity of our system, we selected two economically significant plant RNA viruses: PVX and PVY. We designed multiple RPA primer sets against the coding sequence of the viral CP (Figure 1B, Supplementary Figure 2A, and Supplementary Table S1). We also designed two independent crRNAs, and assessed their CRISPR/Cas12a-based *cis*-cleavage activity against the PCR products amplified from the CP coding sequences. Both crRNAs very efficiently cleaved their intended targets (Supplementary Figure 1A). To confirm the *trans* collateral cleavage activity of CRISPR/Cas12a, we added a single-stranded HEX-labeled DNA reporter to the reaction. In contrast to control samples containing no crRNA, we observed a strong fluorescent signal in the presence of CRISPR/Cas12a. In addition, Cas12a cleaved the ssDNA reporter only when pre-loaded with crRNA (Supplementary Figure 1B).

To test the efficiency of the designed primers, we subjected the PCR products amplified from the CP to *in vitro* transcription, followed by purification and applied to iSCAN-TP detection assay. All primer sets were effective in the iSCAN-TP reaction format, although we noticed that the first two sets were more active (Supplementary Figure 1C). We thus proceeded with the first two primer sets in later PVX RT-RPA experiments (Figure 1C).

To overcome the contamination of the work space problem which may lead to false negative assays, we developed an iSCAN-OP detection assay for plant RNA viruses (RT-RPA-CRISPR/Cas12a). Since the optimal temperature for reverse transcriptase enzymes is typically 42°C or more, we tested whether the Cas12a *cis* and *trans* endonuclease activities might also be active at temperatures other than 37°C. Cas12a exhibited cis and trans activities at all temperatures from 37 to 42°C, that is in line with the previously reported studies (Huang et al., 2020), paving the way for its use for iSCAN-OP detection assay, which we tested next on *in vitro* transcribed PVX-CP. Our detection assay efficiently detected the PVX-CP RNA in a reaction mixture compared to the no template control (NTC) (Figure 1D). To validate our detection system with freshly prepared viral samples, we isolated total RNA from *Nicotiana benthamiana* plants infected with PVX and repeated the iSCAN-OP detection assay. We determined that our detection system also effectively detected viral RNA in total RNA extracted from infected plants (Figure 1E).

Next, we assessed our RT-RPA-CRISPR/Cas12a detection assay against another plant RNA virus, PVY. To this end, we designed two crRNAs targeting the CP coding region of the PVY genome (Supplementary Table S3) and evaluated their activity against a PCR product covering the PVY-CP sequence. The CRISPR/Cas12a-based *cis* cleavage of the PVY-CP PCR product indicated that both crRNAs efficiently directed Cas12a for target cleavage (Supplementary Figure 2B). The addition of the HEX reporter also succeeded in identifying Cas12a target-based collateral activity in the reaction mixture (Supplementary Figure 2C). We measured a strong signal in the reaction mixture compared to NTC, indicating that both crRNAs activated Cas12a *cis* and *trans* cleavage activities. Subsequently, we designed four sets of RPA primers to assess our RT-RPA-CRISPR/Cas12a system against *in vitro*-transcribed PVY-CP RNA. We detected a strong signal in all reactions relative to NTC, with the exception of primer set 4 (S4), where we observed non-specific amplification in the control reaction (Supplementary Figure 2D). This result underscores the need to screen multiple primers sets in isothermal reactions.

### Detection of TMV With the iSCAN-OP Detection Assay

**FIGURE 2:**
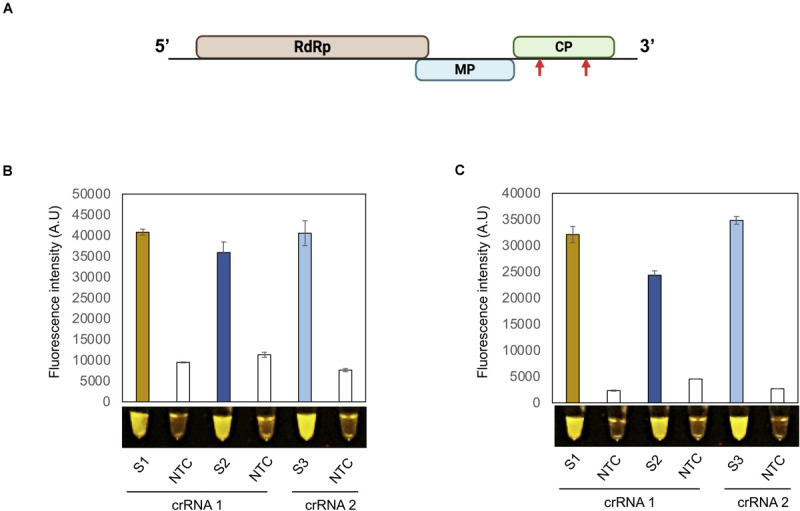
Detection of TMV with the iSCAN-OP detection assay.**(A)** A schematic view of tobacco mosaic virus (TMV) genome design. Red arrows indicate the regions targeted by RT-RPA coupled CRISPR/Cas12a based detection system. **(B)** End-point fluorescence visualization of the iSCAN-OP detection assay (*in vitro* transcribed RNA) with P51 Molecular Fluorescence Viewer. S1: Primer set 1, S2: Primer set 2, S3: primer set 3, NTC: no template control. Values are shown in the graph as means ± SD (*n* = 3). **(C)** End-point fluorescence visualization of the iSCAN-OP detection assay (with plant total RNA) with P51 Molecular Fluorescence Viewer. S1: Primer set 1, S2: Primer set 2, S3: Primer set 3 NTC: no template control. Values are shown in the graph as means ± SD (*n* = 3).

A robust and useful detection assay should be effective in detecting various viruses in addition to PVX and PVY. We therefore selected another plant pathogenic virus with a + ssRNA genome: tobacco mosaic virus (TMV). As described above for PVX and PVY, we designed two crRNAs against the CP coding region of TMV and tested their Cas12a-based *cis* and *trans* cleavage potential (Figure 2A). As shown in Supplementary Figure 3A, a TMV-CP PCR product was efficiently cleaved in a Cas12a/crRNA-dependent manner compared to the control reaction. Both crRNAs also directed Cas12a target-based collateral activity, as evidence by the associated fluorescence signal relative to the control (Supplementary Data 3B).

To generalize the applicability of our detection system, we designed three different RPA primer sets to amplify a region of the TMV-CP and tested their efficacy in an iSCAN-OP detection assay with *in vitro*-transcribed TMV-CP RNA. All primers sets were equally efficient and able to detect TMV-CP-RNA in a iSCAN-OP reaction (Figure 2B). Next, to test the efficiency of these primers on freshly collected samples, we isolated total RNA from *N. benthamiana* plants infected with TMV and subjected them to our detection assay. Again, all primer sets efficiently detected the TMV viral genome when the fluorescent signal was compared to the control reaction (Figure 2C). Collectively, our results suggest that this detection assay can be used and generalized for any plant RNA virus. Such a robust and fast platform will undoubtedly advance the timely detection of any pathogenic plant virus, thus allowing early diagnosis and the implementation of preventive strategies.

### Multiplexing and Sensitivity of the iSCAN-OP Detection Assay

**FIGURE 3:**
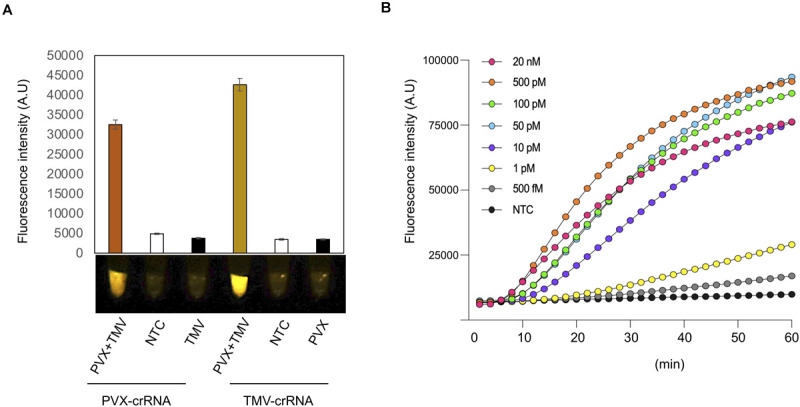
Multiplexing and sensitivity of the iSCAN-OP detection assay. **(A)** End-point fluorescence visualization of the iSCAN-OP detection assay (with plant total RNA) with P51 Molecular Fluorescence Viewer. Values are shown in the graph as means ± SD (*n* = 3). **(B)** Real-time detection of Cas12a trans-cleavage activity using a ssDNA-FQ FAM labeled reporter. Values are shown in the graph as means (*n* = 3). NTC: no template control.

To determine how our iSCAN-OP detection assay performs when plants are infected by more than one virus, we infected 3-week-old *N*. *benthamiana* plants with PVX and TMV, separately or together. We extracted total RNA from plants 7 dai and performed iSCAN-OP detection assays at 42°C for 20 min. We only detected PVX in mixed infection samples when using PVX-specific primers and the appropriate crRNA. We obtained no signal amplification from plants that had been infected only with TMV when using the PVX-specific primers and crRNA, indicating that the observed signal originated from PVX viral RNA exclusively (Figure 3A). Similarly, TMV-specific RPA primers and crRNA only detected TMV viral RNA but not PVX, demonstrating that our detection assay is specific, robust and can be utilized for multiplexed detection of different viruses in situations of mixed infections.

Last, we investigated the detection sensitivity of our RT-RPA-CRISPR/Cas12a detection assay in real-time. To this end, we diluted our *in vitro*-transcribed TMV RNA down to femtomolar concentrations and subjected the serial dilutions to our iSCAN-OP detection assay with the S3 set of RPA primers together with a ssDNA FAM fluorescent reporter to measure fluorescence in real-time. Our results indicate that our CRISPR-based detection assay can detect viral RNA down to the picomolar range, however, longer reaction time can improve the detection limit of the assay down to femtomolar, which is in line with previously reported data (Figure 3B; Cha et al., 2020). However, we noticed that a longer incubation period was required for the effective detection of viral RNA molecules at low concentrations.

## Discussion

Despite various control strategies to prevent viral spread in crop fields, RNA viruses remain a threat to plant health and crop productivity worldwide. Specific and robust detection of plant viruses is crucial for early detection and containment. As the majority of viruses that infect plants harbor RNA genomes, we established a robust and efficient iSCAN-OP detection method combining the RT-RPA and CRISPR/Cas12a-based detection platforms for plant RNA viruses. The development of robust, simple, and affordable detection platforms thus has great potential to lower crop losses. To address this, we combined two methods into such a detection assay: first, RPA amplifies the target sequence at one temperature (isothermal reaction); second, CRISPR/Cas12a collateral nuclease activity is activated in the presence of its target, which was just amplified by RPA. RPA is more convenient and economical than LAMP, as it entails a single pair of primers in comparison to 4–6 primers needed for LAMP. Another advantage of RPA over LAMP and other isothermal amplification methods is its short reaction time (Zaghloul and El-Shahat, 2014).

The collateral activities of several Cas proteins (Cas13, Cas12, and Cas14) have been co-opted to add further specificity in CRISPR/Cas-based diagnostic platforms (Chaijarasphong et al., 2019; Aman et al., 2020; Joung et al., 2020; Kanitchinda et al., 2020). LAMP and RPA are highly robust nucleic acid amplification methods that amplify the target sequence in a much shorter time than traditional PCR. However, the robustness of these methods also constitutes a major limitation of isothermal amplification methods due to amplification of minute amounts of template (oftentimes resulting from cross-contamination), leading to false positives (Nagai et al., 2016; Ali et al., 2020). To overcome these issues, screening more than one pair of primers is recommended to select the best pair. It is also recommended to designate dedicated pre and post-amplification spaces to minimize the chances of cross-contamination (Panno et al., 2020). Isothermal amplification methods have recently been successfully coupled with CRISPR/Cas technology in single-pot reactions to reduce the potential for cross-contamination. The amplification temperature of RPA permits its pairing with CRISPR/Cas12a nuclease activity in a single-pot reaction.

CRISPR-based diagnostics have been used to diagnose different human pathogens. We have recently developed iSCAN assay for specific, sensitive and rapid detection of SARS-CoV-2 (Ali et al., 2020). Here, we report a highly robust and specific iSCAN-OP detection assay for plant RNA viruses; that can detect viral RNA in total RNA extracted from plants in 20 min. Different from the iSCAN assay for SARS-CoV-2, we chose to use RT-RPA instead of RT-LAMP for the development of one-pot assay in iSCAN-OP because RT-RPA and Cas12a detection can be performed in the same temperature.

During optimization of the iSCAN-OP detection assay, we tested the activity of CRISPR/Cas12a at different temperatures and determined that *cis* and *trans*-cleavage mediated by the Cas12a protein was similarly efficient at all tested temperatures, with a slightly higher activity at 42°C. The unequal efficiency of different RPA primers also indicates that all primer sets are not equivalent, pointing to the need to screen several primer sets. Furthermore, we confirmed the efficacy of our iSCAN-OP detection system against several plant RNA viruses, corroborating the efficiency of such systems, not only in plants but also in other species, since their detection principles are independent from the organism. Our assay may be applicable for viroid detection as it has been recently reported that CRISPR-Cas 13a, and 13b have a good potential for application as a rapid and accurate diagnostic assay for known viroids (Hadidi, 2019).

**FIGURE 4:**
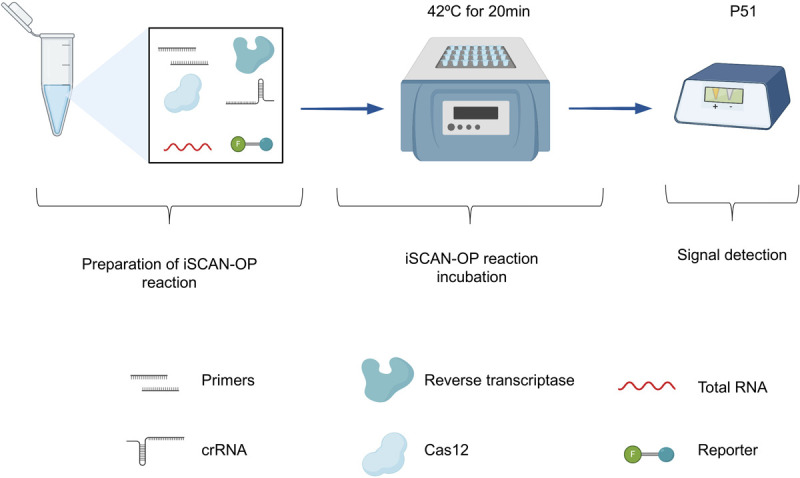
Overview of the iSCAN-OP detection assay. Schematic illustration of the iSCAN-OP detection assay. The diagram describes the addition of all necessary reagents: virus-specific RPA primers, reverse transcriptase, Cas12a protein, crRNA and HEX reporter to the tubes containing the RPA substrate, resuspended from the lyophilized pellet in resuspension buffer. After mixing, total RNA extracted from plant tissue and magnesium acetate is added to the reaction mixture. The reaction mixture is incubated at an isothermal temperature of 42°C for 20 min and the fluorescence signal is measured with the P51 Molecular Fluorescence Viewer.

Our data also suggest that the additional specificity conferred by the CRISPR/Cas assay allows the detection of a specific virus during mixed infections when virus-specific RPA primers are used. We observed strong signals with detecting TMV from plants infected with both TMV and PVX, which we attribute to the high replicative nature of TMV in *N. benthamiana* (relative to PVX). We only detected a single virus in plants infected with TMV or PVX, indicating that the one-pot RT-RPA-CRISPR/Cas12a detection assay is highly specific, and can be clearly adopted for the detection of RNA-based viruses. In addition, the iSCAN-OP detection assay is highly sensitive as we detected TMV synthetic RNA molecules down to femtomolar concentrations. CRISPR/Cas12a can detect RT-RPA-amplified products in 20 min, although a longer incubation time of up to 1 h may be required in the case of low viral titer. Finally, to support in-field diagnosis, we coupled our detection assay with a commercially available and affordable fluorescent reader device (P51 Molecular Fluorescence Viewer). The reaction, incubated at the isothermal temperature of 42°C for 20 min, is transferred to a P51 fluorescent viewer for signal capture (Figure 4). The high specificity of our developed detection assay makes it amenable to be used in mixed infection of two or more viruses.

In conclusion, we have shown that a CRISPR/Cas12a-based detection assay coupled with nucleic acid amplification can detect plant RNA viruses with high specificity and sensitivity. The single-tube detection format contributes to minimizing potential cross-contamination and thus reduces false positives. Moreover, the single temperature requirement of the iSCAN-OP detection assay, together with the freedom from expensive equipment or highly trained personnel, makes this method amenable to be implemented at point-of-care, if coupled with an easy total RNA extraction protocol from plant tissues (Daher et al., 2016). As RPA has been reported to be tolerant to reaction inhibitors compared to other amplification methods such as qPCR, it will be more suitable when coupled with a quick total RNA extraction from plants, as reported for the detection of PVX from *N. benthamiana* (Shahin et al., 2018; Silva et al., 2018). Overall, our developed assay constitutes an efficient and robust detection method that has the potential to be used for RNA plant virus and viroid diagnosis.

## Data Availability Statement

The original contributions presented in the study are included in the article/Supplementary Material, further inquiries can be directed to the corresponding author/s.

## Author Contributions

MM conceived the research, analyzed the data, and managed the project. RA, AM, TM, and NH designed the research. RA and AM performed the research. RA, AM, and MM wrote the manuscript. All authors contributed to the article and approved the submitted version.

## Conflict of Interest

The authors declare that the research was conducted in the absence of any commercial or financial relationships that could be construed as a potential conflict of interest.

## Footnotes

1 https://www.minipcr.com/product/p51-molecular-glow-lab/
