# Supplementary material for: Efficient, Rapid, and Sensitive Detection of Plant RNA Viruses With One-Pot RT-RPA–CRISPR/Cas12a Assay
Source: Front Microbiol. 2020 Dec 17;11:610872. doi: 10.3389/fmicb.2020.610872 (PMC7773598; doi:10.3389/fmicb.2020.610872)
Supplement: Supplementary file 1 [file Data_Sheet_1.PDF]

Supplementary data file

Efficient, rapid and sensitive detection of plant RNA viruses with one-pot RT-RPA–CRISPR/Cas12a assay

Rashid Aman, Ahmed Mahas, Tin Marsic, Norhan Hassan, and Magdy M. Mahfouz †

Laboratory for Genome Engineering and Synthetic Biology, Division of Biological Sciences, 4700 King Abdullah University of Science and Technology, Thuwal 23955-6900, Saudi Arabia.

†**Correspondence**: Magdy M. Mahfouz (magdy.mahfouz@kaust.edu.sa)

Keywords: RT-RPA, CRISPR-Cas12a, plant virus RNA, biosensors, diagnostics

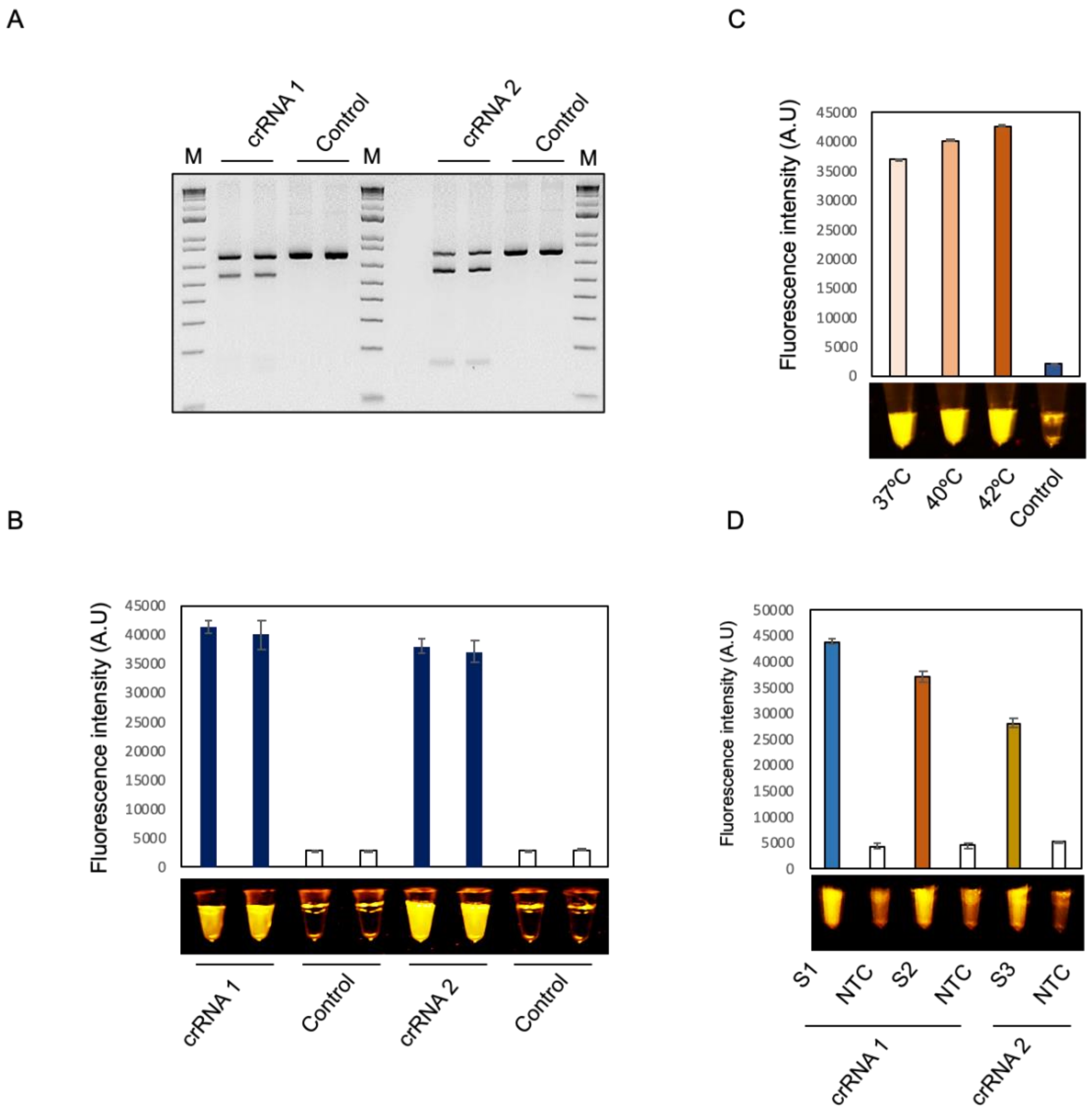

Supplementary Figure 1. Assessment of enzyme activity and screening of RPA primer sets.

A) Cas12a-based restriction of target dsDNA. Visualization of multiple fragments generated by Cas12a *cis*-nuclease activity of the target regions by agarose gel electrophoresis. M: 1 kb plus DNA ladder. Control: no crRNA.

B) Fluorescence signal visualization of CRISPR/Cas12a *trans* cleavage activity with the P51 Molecular Fluorescence Viewer. Data are shown as mean ±SD (*n*=3).

C) Fluorescence signal visualization of CRISPR/Cas12a *trans* cleavage activity at different temperatures. Data are shown as mean ±SD (*n*=3).

D) Screening of different PVX RPA primer sets with iSCAN-OP detection system. S1: Primer set 1, S2: Primer set 2, S3 Primer set 3, NTC: no template control. Data are shown as mean ±SD (*n*=3).

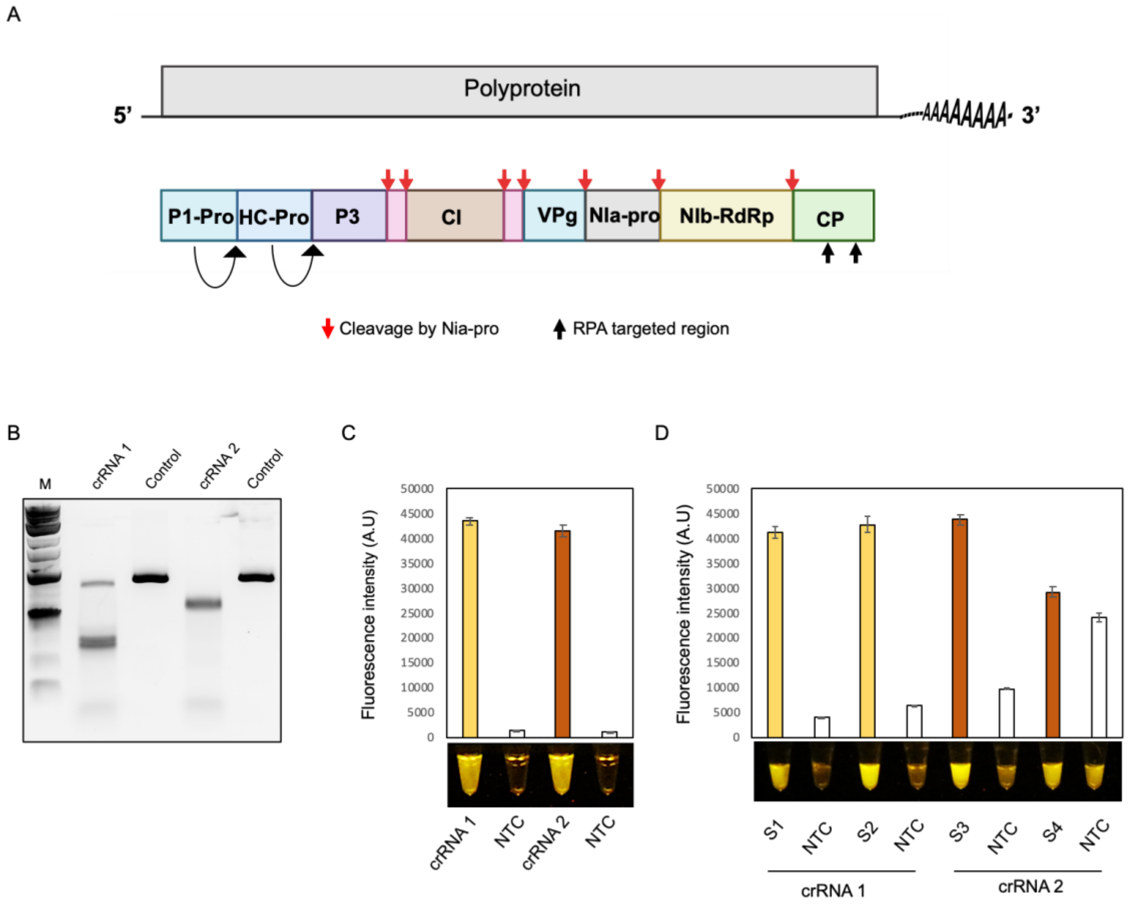

Supplementary Figure 2. PVY detection with the iSCAN-OP detection assay

A. A schematic overview of Potato virus Y (PVY) genome design. Black arrows indicate the regions targeted by the RT-RPA-CRISPR/Cas12a detection system. Red arrows indicate the cleavage of the polyprotein by NIa-pro.
B. Cas12a-based restriction of target dsDNA. Visualization of multiple fragments generated by Cas12a *cis*-nuclease activity of the target regions by agarose gel electrophoresis. M: 2-log DNA ladder. Control: no crRNA.
C. Fluorescence signal visualization of CRISPR/Cas12a trans cleavage activity with P51 Molecular Fluorescence Viewer. NTC: no template control. Data are shown as mean ±SD (*n*=3).
D. Screening of different PVY RPA primer sets for the iSCAN-OP detection system. S1: Primer set 1, S2: Primer set 2, S3 Primer set 3, S4: Primer set 4, NTC: no template control. Data are shown as mean ±SD (*n*=3).

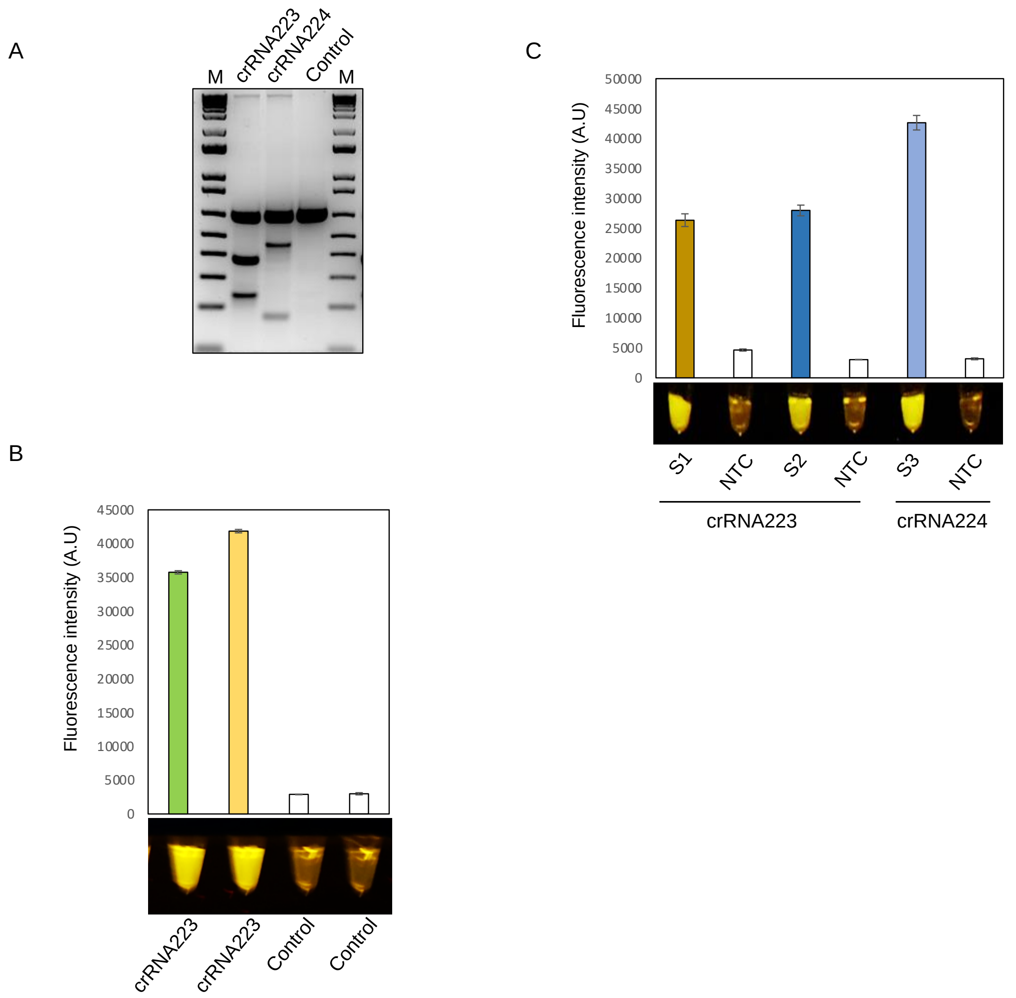

Supplementary Figure 3. Activity assessment of TMV crRNAs and screening of RPA primers.

A. Cas12a-based restriction of target dsDNA. Visualization of multiple fragments generated by Cas12a *cis*-nuclease activity of the target regions by agarose gel electrophoresis. M1: 2-log DNA ladder. M2: 1 kb plus DNA ladder, Control: no crRNA.
B. Fluorescence signal visualization of CRISPR/Cas12a *trans* cleavage activity with the P51 Molecular Fluorescence Viewer. NTC: no template control. Data are shown as mean ±SD (*n*=3).
C. Screening of different TMV RPA primer sets in the iSCAN-OP detection system. S1: Primer set 1, S2: Primer set 2, S3 Primer set 3, NTC: no template control. Data are shown as mean ±SD (*n*=3).

Supplementary Table S1. Primers used for *in vitro* transcription and PCR amplification of target genes.

**Primer Name**	**Sequence, 5`–3`**	**Purpose**
PVX-CP-T7-F	GAAATTAATACGACTCACTATAGGGatgtcagcaccagctagcacaacacag	To PCR amplify PVX-CP region for *cis*-cleavage activity assessment and *in vitro* transcription
PVX-CP-R	ttatggtggtggtagagtgacaacagc	
PVY-CP-T7-F	GAAATTAATACGACTCACTATAGGGGCAAATGACACAATTGATGCAGGAGGAAGC	To PCR amplify PVY-CP region for *cis*-cleavage activity assessment and *in vitro* transcription
PVY-CP-R	CATGTTCTTGACTCCAAGTAGAGTATGC	
TMV-CP-T7-F	GAAATTAATACGACTCACTATAGGGATGTCTTACAGTATCACTACTCCATC	To PCR amplify PVY-CP region for *cis*-cleavage activity assessment and *in vitro* transcription
TMV-CP-R	CGTGCCTGCGGATGTATATGAACC	

Supplementary Table S2. LbCas12a crRNAs

Cas Enzyme	Sequence (5`–3`)	Genomic Region
T7-3G IVT primer-1	GAAATTAATACGACTCACTATAGGG	Anneals with all crRNAs
crRNA-Cas12a-PVX-CP-1-R	tccggccaccgtctgaagctATCTACACTTAGTAGAAATTACCCTATAGTGAGTCGTATTAATTTC	PVX-CP region
crRNA-Cas12a-PVX-CP-2-R	atggcacgggctgtactaaaATCTACACTTAGTAGAAATTACCCTATAGTGAGTCGTATTAATTTC	PVX-CP region
crRNA-Cas12a-PVY-CP-1-R	GGCTTATGGTTTGGTGCATTATCTACACTTAGTAGAAATTACCCTATAGTGAGTCGTATTAATTTC	PVY-CP region
crRNA-Cas12a-PVY-CP-2-R	GGGCTAGGGAAGCGCAAATTATCTACACTTAGTAGAAATTACCCTATAGTGAGTCGTATTAATTTC	PVY-CP region
crRNA-Cas12a_TMV-CP-1-R	CGCATTGTACCTGTACACCTATCTACACTTAGTAGAAATTACCCTATAGTGAGTCGTATTAATTTC	TMV-CP region
crRNA2-Cas12a_TMV-CP-2-R	ACCAAACCAGAAGAGCTCTCATCTACACTTAGTAGAAATTACCCTATAGTGAGTCGTATTAATTTC	TMV-CP region

Supplementary Table S3. RPA Primers

Primer Name	Sequence, 5`–3`	Set	Targeted region
RPA-PVX-CP-2-R	cgttggattgtgccctggcctttgtaatcttc	Primer set 1 (S1)	PVX coat protein
RPA-PVX-CP-2-F	gacttcttcaatggagtcaccaacccagctgc		
RPA-PVX-CP-2-R2	gagtgacagctgcatctaggctggcaaagt	Primer set 2 (S2)	PVX coat protein
RPA_PVX-CP-2-F2	cccagctgccatcatgcccaaagaggggctc		
RPA-PVX-CP-3-F	caactacctcaactaccacaaaaactgcaggc	Primer set 3 (S3)	PVX coat protein
RPA-PVX-CP-3-R	ccttccaaatagcctcaatcttgctgaggtcctC		
RPA-PVX-CP-2-R	ACCCGAAAAGTCGAGGTTGAGCTGATTTCAATG	Primer set 1 (S1)	PVY coat protein
RPA-PVY-CP-1-F	GCATACGACATAGGAGAAACTGAGATGCCAAC		
RPA-PVY-CP-1-F2	GAAGCAGTGCGGATGGCATACGACATAGGAG	Primer set 2 (S2)	PVY coat protein
RPA-PVY-CP-1-R	GTTGGTTTTGCATTCTCAACGATTGGTTTCA		
RPA-PVY-CP-1-R2	CTCAACGATTGGTTTCAACGGGTACTCAACTTG	Primer set 3 (S3)	PVY coat protein
RPA-PVY-CP-2-F	GAAATGCGCAACAAAAAGGAACCATATATGCCAC		
RPA-PVY-CP-2-F2	CAACAAAAAGGAACCATATATGCCACGATATGG	Primer set 4 (S4)	PVY coat protein
RPA-PVY-CP-2-R2	GTACTGATGCCACCGTCCAACCCGAAAAGTC		
TMV-RT-RPA-F1	CACCACAAGTAACTGTTAGGTTCCCTGACAG	Primer set 1 (S1)	TMV coat protein
TMV-RT-RPA-R1	GTCGAATGCACCTAACAGTGCTGTGACTAGC		
TMV-RT-RPA-F2	GACAATTCAGTGAGGTGTGGAAACCTTCAC	Primer set 2 (S2)	TMV coat protein
TMV-RT-RPA-R2	GTGGGGTTCGCCTGATTTTCAACTTCTATTATTC		
TMV-RT-RPA-F3	GAATTGATCAGAGGAACCGGATCTTATAATCGG	Primer set 3 (S3)	TMV coat protein
TMV-RT-RPA-R3	CATCTTGACTACCTCAAGTTGCAGGACCAG		
